# Supplementary material for: Optimal multisensory decision-making in a reaction-time task
Source: eLife. 2014 Jun 14;3:e03005. doi: 10.7554/eLife.03005 (PMC4102720; doi:10.7554/eLife.03005)
Supplement: Supplementary file 1. — Detailed model derivation and description. DOI: http://dx.doi.org/10.7554/eLife.03005.015 [file elife03005s001.pdf]

Optimal multisensory decision-making in a reaction-time task

Detailed model derivation and description

Jan Drugowitsch, Gregory C. DeAngelis, Eliana M. Klier,
Dora E. Angelaki, Alexandre Pouget

Optimal Accumulation of Evidence across Time and Cues

The subjects are seated on a motion platform in front of a display screen. They receive information about self-motion direction either through vestibular cues (platform motion), visual cues (visual flow field), or through both cues in combination. They perform a heading discrimination task, and are instructed to indicate as quickly and as accurately as possible if they are moving rightward or leftward relative to straight ahead. Below, we develop – based on a combination of Bayesian inference and diffusion models – an ideal observer model for this task.

Denote h as the heading direction, with $h=0$ being straight ahead, and $h>0$ being rightward motion relative to straight ahead. Each sensory cue provides, at each point in time, noisy momentary evidence about this heading direction. The task of the decision maker is to accumulate this evidence over time and cues, and, after some time, decide whether $h \geq 0$ or $h<0$ based on the belief:

$$p\left(h \geq 0 \mid \text{all momentary evidence}\right). \tag{1}$$

Below, we first describe for each cue how we assume this momentary evidence to encode h, and then we show how to compute the above belief for various, increasingly realistic cases. We begin by assuming a single sensory cue that provides evidence whose reliability is constant over time. This leads to a formulation similar to diffusion models (Ratcliff 1978; Bogacz, Brown et al. 2006), and we utilize this relationship to model the

speed/accuracy trade-off. We then show how to extend this formulation to cases in which the reliability of the momentary evidence changes with time, as is the case in both of our unimodal conditions. After that, we derive how a single diffusion model can optimally accumulate evidence across cues, even if the reliability of the evidence differs between the cues. We will again initially assume the reliability of the evidence to be constant in time, and then deal with the general case of time-varying cue reliability in which the time-course differs between the two cues. The last case describes the ideal observer/actor model for the multi-modal condition of our task.

In what follows, the subscripts $\cdot_{vis}$, $\cdot_{vest}$, and $\cdot_{comb}$ refer to the visual-only, vestibular-only, and combined conditions, respectively.

Momentary Evidence

We assume that, for either modality, information about heading direction is encoded by a noisy sensory signal, called the *momentary evidence* and denoted by $\dot{x}$, according to

$$\dot{x} = b(t)k\sin(h) + \eta(t), \tag{2}$$

where $b(t)k$ is the *sensitivity* that determines the strength with which h influences $\dot{x}$, and $\eta(t)$ is a unit variance Gaussian white noise process. The only information about heading relevant to our task is the horizontal projection onto a line that is orthogonal to straight ahead. For this reason, heading h influences the momentary evidence only through this projection, as given by $\sin(h)$.

The subject's sensitivity to the evidence is characterized by the terms k and $b(t)$, where k determines how effectively each subject can make use of the incoming information, and $b(t)$ encodes how the reliability of this information changes over time. Thus, the sensitivity $b(t)k$ is a composite measure that takes into account both how reliably the momentary evidence $\dot{x}$ reveals the heading direction (determined by experimenter), as well as how effective the subject is in using this information (property of subject). Acceleration, $a(t)$, is the physical quantity that modulates the reliability of the inertal motion (i.e, vestibular) cues, such that the subject's sensitivity follows the same time course, $b_{vest}(t) = a(t)$. We assume that, at least for the slow speeds used in the experiment, the reliability of the visual cue is mainly determined by motion velocity,

$v(t)$, such that $b_{vis}(t) = v(t)$ (Lisberger and Movshon 1999; Price, Ono et al. 2005; Schlack, Krekelberg et al. 2007).

For further development, we consider a time-discretized version of the momentary evidence, δx_n, that is related to $\dot{x}$ by $\delta x_n = \int_{n\Delta}^{(n+1)\Delta} \dot{x}(\mathrm{t})\mathrm{d}t$, where Δ denotes the short time periods into which time is discretized. Given that Δ is sufficiently small, we can assume the reliability time-course $b_n \approx b(n\Delta)$ to be constant for each n. Then, $\delta x_n \sim \mathrm{N}(kb_n \sin(h)\Delta, \Delta)$ is distributed according to a normal distribution with mean $kb_n \sin(h)\Delta$ and variance Δ. As $\Delta \to 0$, the use of either x or δx_n becomes equivalent. Thus, all of the following is valid for either definition. The amount of information that δx_n provides for the discrimination of $\sin(h)$, as measured by the Fisher Information, is given by $\mathrm{I}_{\delta x_n}(\sin(h)) = k^2 b_n^2 \Delta$. This confirms kb_n as measure of sensitivity to changes in the momentary evidence.

Accumulating Evidence with Constant Reliability over Time

Assume that the subject observes the stimulus for T seconds and wants to decide between $h < 0$ and $h \geq 0$ based on all momentary evidence observed up to that point. For now we assume the reliability of the evidence to be constant over time, such that $\forall t \geq 0 : b(t) = 1$ and the momentary evidence becomes $\delta x_n \sim \mathrm{N}\left(k \sin(h)\Delta, \Delta\right)$.

Given that $h \geq 0$ corresponds to $\sin(h) \geq 0$ over the range of headings of interest, we want to find the belief $p(\sin(h) > 0 \mid \delta x_{1:N})$, where $N \approx T/\Delta$ and $\delta x_{1:N} = \{\delta x_1, \ldots, \delta x_N\}$ denotes all momentary evidence up to time $T \approx \Delta N$. In order to do so, we first compute the posterior $\sin(h)$ by Bayes' rule, resulting in

$$
\begin{aligned}
p\left(\sin(h) \mid \delta x_{1:N}\right) &\propto \prod_{n=1}^{N} p\left(\delta x_n \mid \sin(h)\right) \\
&= \prod_{n=1}^{N} \mathrm{N}\left(\delta x_n \mid k\sin(h)\Delta, \Delta\right) \\
&\propto e^{\sin(h)k\sum_n \delta x_n - \frac{1}{2}\sin(h)^2 k^2 \sum_n \Delta} \\
&\approx e^{\sin(h)kx(T) - \frac{1}{2}\sin(h)^2 k^2 T} \\
&\propto \mathrm{N}\left(\sin(h) \mid \frac{x(T)}{kT}, \frac{1}{k^2 T}\right),
\end{aligned}
\tag{3}
$$

where we have assumed a uniform prior, $p(\sin(h)) \propto 1$ over $\sin(h) \in [-1,1]$, and have used $\sum_n \delta x_n \approx \int_0^T \dot{x}(t)\mathrm{d}t = x(T)$ and $\sum_n \Delta \approx T$. Furthermore, we have assumed that $|\sin(h)|$ is small (as is the case in our experiment, with $|h| \leq 16\,\mathrm{deg}$ and thus $|\sin(h)| < 0.276$), such that we can approximate the posterior by a Gaussian despite the restriction that $\sin(h) \in [-1,1]$. The above shows that the posterior only depends on the sufficient statistics $x(T)$ and T, rather than the whole sequence $\delta x_{1:N}$ of observations. Furthermore, the posterior variance decreases monotonically with T, as more evidence provides us with a more certain estimate.

From this posterior we find the belief of $\sin(h) \geq 0$ by

$$
p\left(\sin(h) \geq 0 \mid x(T), T\right) = \int_0^1 p\left(\sin(h) = y \mid x(T), T\right)\mathrm{d}y \approx \Phi\left(\frac{x(T)}{kT}\sqrt{k^2 T}\right) = \Phi\left(\frac{x(T)}{\sqrt{T}}\right), \tag{4}
$$

where we again have assumed the posterior to be well approximated by a Gaussian that has negligible mass outside the range $[-1,1]$, and $\Phi(\alpha) = \int_{-\infty}^{\alpha} N(\beta \mid 0,1)\mathrm{d}\beta$ is the standard cumulative Gaussian, with $\Phi(\alpha) \geq \frac{1}{2}$ if $\alpha \geq 0$ and $\Phi(\alpha) < \frac{1}{2}$ otherwise. Consequently, the decision maker ought to decide in favor of $h \geq 0$ if $x(T) \geq 0$, and $h < 0$ otherwise (Drugowitsch, Moreno-Bote et al. 2012).

The above describes the optimal decision strategy given all observed momentary evidence up to some time T. However, it does not provide us with a way to choose at which time enough evidence has been collected to commit to a decision. We achieve the latter by linking this decision strategy to standard diffusion models in the next section.

Relation to Standard Diffusion Models

The framework described above can be cast as a standard diffusion model, in which accumulated momentary evidence is represented by the position of a drifting and diffusing “particle”, $x(t)$. Decisions are triggered by bounding the particle space from below at $-\theta$ and from above by θ. As soon as either of these bounds is reached, the decision maker ought to commit to the corresponding decision. Thus, diffusion models provide us with a strategy for choosing at which time to commit to a decision. Furthermore, the analysis in the previous section shows that these decisions take into account all momentary evidence up until the point of the decision and are therefore Bayes-optimal (Laming 1968; Gold and Shadlen 2002; Bogacz, Brown et al. 2006).

From the perspective of diffusion models it seems as if the only decisive factor to commit to a decision is the particle location $x(t)$. In contrast, our Bayesian analysis above seems to additionally require information about time, t, to compute the relevant probabilities. The omission of time in diffusion models stems from partitioning the belief space into $p\left(\sin(h) \geq 0 \mid x(T), T\right) \geq \frac{1}{2}$ and $p\left(\sin(h) \geq 0 \mid x(T), T\right) < \frac{1}{2}$ while neglecting the actual magnitude of this belief. This magnitude, however, is informative about the certainty at which this decision is made. Consider, for example, that the decision maker chooses $h \geq 0$ at time t, corresponding to $x(t) = \theta$. Then, the belief of making a correct decision is given by

$$p\left(\sin(h) \geq 0 \mid x(t) = \theta, t\right) = \Phi\left(\frac{\theta}{\sqrt{t}}\right) \geq \frac{1}{2}, \tag{5}$$

which is a decreasing function of time. This expression follows directly from Eq. (4) despite the presence of a bound, as we (implicitly) condition on having reached the bound for the first time at time t, such that we do not need to consider the possibility of having crossed this bound before (Drugowitsch, Moreno-Bote et al. 2012). This demonstrates that, even with a constant bound in particle space, diffusion models make decisions at different levels of confidence (Kiani and Shadlen 2009). In particular, early decisions will be of high confidence, while late decisions are made at a low level of confidence. Thus, this constant bound in particle space corresponds to a collapsing bound in belief space.

Qualitatively, such a strategy has been shown to perform optimal decision-making in the sense of maximizing the reward rate (Drugowitsch, Moreno-Bote et al. 2012).

When defining the momentary evidence about heading, we have followed standard diffusion model conventions and have assumed a unit diffusion variance. We will show that this is not a restriction, as for any diffusion model with a non-unit variance we can find a diffusion model with unit variance that features exactly the same behavior. Therefore, we can assume unit variance without a loss of generality. In particular, assume a diffusion variance σ^2, such that the momentary evidence becomes $\dot{x}_\sigma = b(t)k_\sigma \sin(h) + \sigma\eta(t)$. This evidence relates to the unit-variance evidence by $\dot{x} = \dot{x}_\sigma / \sigma$ with $k = k_\sigma / \sigma$. The same relationship $x(t) = x_\sigma(t) / \sigma$ holds between the particle locations (i.e. the accumulated momentary evidence). This shows that assuming a non-unit variance is equivalent to a re-scaling of the particle space. We can compensate for this re-scaling by re-scaling the bounds, leading to a diffusion model with exactly the same behavior as one with a unit diffusion variance. We will use this property later, to find the minimal parameterization of our model while assuming that the diffusion variance is modulated by visual motion coherence.

Accumulating Evidence with Time-Varying Reliability

In this section we will show that, as soon as the reliability of the evidence changes over time, particle location and time are no longer sufficient statistics. Instead, we need to take the changing reliability of the cues into account when accumulating the momentary evidence. This will lead to a re-definition of the particle location in diffusion models that allows us to make Bayes-optimal decisions even with time-varying reliability of the momentary evidence.

As before, we are interested in computing the belief $p\left(\sin(h) \geq 0 \mid \delta x_{1:N}\right)$. However, now we assume the sensitivity k to be weighted by the time-varying function $b(t)$, such that the momentary evidence is given by $\delta x_n \sim N\left(kb_n \sin(h)\Delta, \Delta\right)$. With this evidence, the posterior of $\sin(h)$ results in

$$
\begin{aligned}
p\left(\sin(h) \mid \delta x_{1:T}\right) &\propto \prod_{n=1}^{N} \mathrm{N}\left(\delta x_n \mid k b_n \sin(h)\Delta, \Delta\right) \\
&\propto e^{\sin(h) k \sum_n b_n \delta x_n - \frac{1}{2}\sin(h)^2 k^2 \sum_n b_n^2 \Delta} \\
&\approx e^{\sin(h) k X(T) - \frac{1}{2}\sin(h)^2 k^2 B(T)} \\
&\propto N\left(\sin(h) \mid \frac{X(T)}{kB(T)}, \frac{1}{k^2 B(T)}\right),
\end{aligned}
\tag{6}
$$

where we have defined

$$
X(T) = \int_0^T b(t)\dot{x}(t)\mathrm{d}t \approx \sum_n b_n \delta x_n, \quad B(T) = \int_0^T b(t)^2 \mathrm{d}t \approx \sum_n b_n^2 \Delta. \tag{7}
$$

Thus, $X(T)$ is the accumulated momentary evidence, weighted at each point in time by the sensitivity time course. $B(T)$ is the squared accumulated sensitivity time course, which we will call the *power* of the evidence. Comparing Eq. (3) to Eq. (6) shows that $X(T)$ replaces $x(T)$ as the particle location, and $B(T)$ becomes the new passed time, replacing T. Using $b(t) = 1$ for all t causes $B(T) = T$ and $X(T) = x(T)$ and recovers the original formulation. While it might seem that a negative $b(t)$ (for example, in the case of acceleration) causes the momentary evidence in Eq. (7) to be weighted negatively, this is in fact not the case, as $\dot{x}(t)$ is already scaled by $b(t)$ according to Eq. (2). Thus, if we replace $\dot{x}(t)$ in Eq. (7) by Eq. (2), $b(t)$ will be squared, causing its effective influence on the momentary evidence to be always non-negative.

With the above posterior, the belief becomes

$$
p(\sin(h) \geq 0 \mid X(T), B(T)) = \Phi\left(\frac{X(T)}{\sqrt{B(T)}}\right). \tag{8}
$$

Therefore, the sign of $X(T)$ now determines the decision. This confirms that $X(t)$ takes the role of the particle location in a Bayes-optimal diffusion model that triggers a decision as soon as either of the bounds is reached.

The above derivation shows that diffusion models that use the un-weighted $x(t)$ as their particle location become sub-optimal as soon as the reliability of the evidence changes with time. Consider, for example, a task in which $b(t) = 0$ for $0 \leq t \leq T$, and $b(t) = 1$ for $t > T$, such that for all $t \leq T$, the momentary information contains only noise and no information about h. If we were to use $x(t)$, we would initially only accumulate

noise while treating it as evidence, which is clearly sub-optimal. $X(t)$ avoids this problem by giving zero weight to all evidence up until T, and only starts accumulating evidence thereafter. This principle finds its parallel in the standard cue combination literature, where it is known that cues ought to be weighted according to their reliability (Clark and Yuille 1990). If one of the cues has a very low reliability, it does not contribute to the decision. The same applies here, but rather than accumulating evidence across cues, we accumulate across time.

Using $X(T)$ instead of $x(T)$ and $B(T)$ instead of T requires a re-interpretation of what a time-invariant bound on $X(T)$ means. From Eq. (8) we can see that the decision confidence at the bound (where $X(T)=\theta$) drops monotonically with $B(T)$. Thus, a constant bound on the particle location still implies a collapsing bound on belief (as $B(T)$ is monotonically increasing in time), but that the latter drops with $B(T)$ rather than with T. Thus, the rate at which this bound drops now depends on the reliability of the momentary evidence. This completes the description of the Bayes-optimal decision making model for the two unimodal conditions.

Accumulating Evidence across Time and Cues, with Constant Reliability

We now describe how to accumulate momentary evidence if information about heading direction is available from multiple cues. For now, we assume the reliability of these cues to be constant over time. In the next section, we discuss the changes required when this is not the case.

The visual and vestibular cues to heading provide momentary evidence given by $\delta x_{vis,n} \sim \mathrm{N}\left(k_{vis}\sin(h)\Delta,\Delta\right)$ and $\delta x_{vest,n} \sim N\left(k_{vest}\sin(h)\Delta,\Delta\right)$. Here, the sensitivities k_{vis} and k_{vest} are again composite measures of how much information the momentary evidence provides about the heading, and how effective subjects are in utilizing this information. Given $\delta x_{vis,1:N}$ and $\delta x_{vest,1:N}$ up until time $T \approx N\Delta$, we find the posterior over $\sin(h)$ by Bayes rule, resulting in

$$p\left(\sin(h) \mid \delta x_{vis,1:N}, \delta x_{vest,1:N}\right) \propto \prod_n \mathrm{N}\left(\delta x_{vis,n} \mid k_{vis}\sin(h)\Delta, \Delta\right) N\left(\delta x_{vest,n} \mid k_{vest}\sin(h)\Delta, \Delta\right)$$
$$\propto e^{\sin(h)\left(k_{vis}\sum_n \delta x_{vis,n} + k_{vest}\sum_n \delta x_{vest,n}\right) - \frac{1}{2}\sin(h)^2\left(k_{vis}^2 + k_{vest}^2\right)\sum_n \Delta}$$
$$\approx e^{\sin(h)\left(k_{vis}x_{vis}(T) + k_{vest}x_{vest}(T)\right) - \frac{1}{2}\sin(h)^2\left(k_{vis}^2 + k_{vest}^2\right)T} \tag{9}$$
$$\propto \mathrm{N}\left(\sin(h) \mid \frac{x_{comb}(T)}{k_{comb}T}, \frac{1}{k_{comb}^2 T}\right),$$

where we have used $\sum_n \delta x_{vis,n} \approx x_{vis}(T)$, $\sum_n \delta x_{vest,n} \approx x_{vest}(T)$, and $\sum_n \Delta \approx T$, and have defined

$$k_{comb}^2 = k_{vis}^2 + k_{vest}^2, \quad x_{comb}(T) = \frac{k_{vis}}{k_{comb}} x_{vis}(T) + \frac{k_{vest}}{k_{comb}} x_{vest}(T). \tag{10}$$

This shows that the sensitivity, k_{comb}, based on both cues is at least as large as that of the more reliable cue, such that $k_{comb} \geq \max\{k_{vis}, k_{vest}\}$. Furthermore, the combined particle location is a weighted sum of the particle locations for the two cues, with weights proportional to the sensitivity to either cue. The decision maker's belief regarding $\sin(h) \geq 0$ is again given by Eq. (4), with $x(T)$ replaced by $x_{comb}(T)$.

Accumulating Evidence across Time and Cues, with Time-Varying Reliability

In our task, the reliability of both cues varies over time. Furthermore, the time-course of variations in reliability differs between the two cues. As previously described, we assume the momentary evidence of the visual modality to be $\delta x_{vis,n} \sim \mathrm{N}\left(k_{vis}v_n sin(h)\Delta, \Delta\right)$, and that of the vestibular modality to be $\delta x_{vest,n} \sim \mathrm{N}\left(k_{vest}a_n sin(h)\Delta, \Delta\right)$, where v_n and a_n denote stimulus velocity and acceleration, respectively. Making use of momentary evidence $\delta x_{vis,1:N}$ and $\delta x_{vest,1:N}$ until time $T \approx N\Delta$, the posterior over $\sin(h)$ becomes

$$p\left(\sin(h) \mid \delta x_{vis,1:N}, \delta x_{vest,1:N}\right) \propto e^{\sin(h)\left(k_{vis}\sum_n v_n \delta x_{vis,n} + k_{vest}\sum_n a_n \delta x_{vest,n}\right) - \frac{1}{2}\sin(h)^2\left(k_{vis}^2\sum_n v_n^2\Delta + k_{vest}^2\sum_n a_n^2\Delta\right)}$$
$$\approx e^{\sin(h)\left(k_{vis}X_{vis}(T) + k_{vest}X_{vest}(T)\right) - \frac{1}{2}\sin(h)^2\left(k_{vis}^2 V(T) + k_{vest}^2 A(T)\right)} \tag{11}$$
$$\propto \mathrm{N}\left(\sin(h) \mid \frac{X_{comb}(T)}{k_{comb}D(T)}, \frac{1}{k_{comb}^2 D(T)}\right).$$

To derive the above, we have, as in Eq. (7), defined the sensitivity-weighted accumulated momentary evidence for each of the cues,

$$X_{vis}(T)=\int_0^T v(t)\dot{x}_{vis}(t)\mathrm{d}t \approx \sum_n v_n \delta x_{vis,n}, \quad X_{vest}(T)=\int_0^T a(t)x_{vest}(t)\mathrm{d}t \approx \sum_n a_n \delta x_{vest,n}, \quad (12)$$

and the accumulated power of the evidence for each cue,

$$V(T)=\int_0^T v(t)^2 \mathrm{d}t \approx \sum_n v_n^2 \Delta, \quad A(T)=\int_0^T a(t)^2 \mathrm{d}t \approx \sum_n a_n^2 \Delta. \quad (13)$$

Furthermore, we have left the definition of k_{comb} unchanged (see Eq. (10)), such that the total power of the evidence is given by

$$D(T)=\frac{k_{vis}^2}{k_{comb}^2}V(T)+\frac{k_{vest}^2}{k_{comb}^2}A(T). \quad (14)$$

As a consequence, the particle location for the combined diffusion model is, similar to Eq. (10), given by

$$X_{comb}(T)=\frac{k_{vis}}{k_{comb}}X_{vis}(T)+\frac{k_{vest}}{k_{comb}}X_{vest}(T), \quad (15)$$

This shows that, even if we have multiple sources of evidence whose reliability varies independently over time, we can express the process of accumulating evidence in a single diffusion model, defined by particle location $X_{comb}(t)$, with $D(t)$ being the quantity that represents the passage of time.

Based on the above formulation, we can derive how the momentary evidence of the combined diffusion model is constructed from the momentary evidence provided by the two cues. If we use $D(t)=\int_0^t d(s)^2 \mathrm{d}s$, and replace $V(t)$ and $A(t)$ in Eq. (14) by their respective definitions in Eq. (13), we find the momentary power of the combined evidence to be given by

$$d(t)^2=\frac{k_{vis}^2}{k_{comb}^2}v(t)^2+\frac{k_{vest}^2}{k_{comb}^2}a(t)^2, \quad (16)$$

which is the sensitivity-weighted average of the momentary powers of the two cues. With the above, Eq. (15) shows that the combined momentary evidence, $\dot{x}_{comb}(t)$, as used in $X_{comb}(t)=\int_0^t d(s)\dot{x}_{comb}(s)\mathrm{d}s$, is composed of

$$\dot{x}_{comb}(t) = \frac{k_{vis}v(t)}{k_{comb}d(t)}\dot{x}_{vis}(t) + \frac{k_{vest}a(t)}{k_{comb}d(t)}\dot{x}_{vest}(t), \quad (17)$$

that is, the sensitivity-weighted sum of the momentary evidence of each of the cues. With these two quantities, the momentary evidence in the combined diffusion model is given by $\dot{x}_{comb} = k_{comb}d(t)\sin(h) + \eta(t)$. This is easily verified by replacing $\dot{x}_{vis}(t)$ and $\dot{x}_{vest}(t)$ in Eq. (17) by their definitions, which leads to the above expression.

To obtain the belief regarding $\sin(h) \geq 0$, we again take the integral of the posterior $\sin(h)$ over $\sin(h) \geq 0$, which gives

$$p\left(\sin(h) \geq 0 \mid X_{comb}(T), D(T)\right) = \Phi\left(\frac{X_{comb}(T)}{\sqrt{D(T)}}\right). \quad (18)$$

Thus, as before, belief depends on particle location $X_{comb}(T)$ and accumulated power $D(T)$, and the decision itself is solely determined by the sign of $X_{comb}(T)$. As a consequence, we can again perform Bayes-optimal decision making by assuming bounds on $X_{comb}(t)$ at $-\theta$ and θ, and decide in favor of $\sin(h) \geq 0$ (or $\sin(h) < 0$) when the particle reaches the upper (or lower) bound. This completes the description of the Bayes-optimal decision model for the combined condition.

Optimality of evidence accumulation

The above describe how to perform Bayes-optimal decision making in different scenarios. Bayes-optimal here means that the posterior upon which the decision is based contains the information of all momentary evidence observed from either cue. This is easily demonstrated in terms of preservation of information. Formally, if $I_{\delta_x}(\sin(h))$ denotes the Fisher information that δ_x provides about changes in $\sin(h)$, then the Fisher information in the posterior (e.g. Eq. (11)) is the sum of the Fisher information of all momentary evidence across both time and cues, that is

$$
\begin{aligned}
\mathrm{I}_{X_{comb}(T)}\left(\sin(h)\right) = k_{comb}^2 D(T) & \\
&= k_{vis}^2 V(T) + k_{comb}^2 A(T) \\
&= \sum_{n=0}^{N}\left(k_{vis}^2 v_n^2 \Delta + k_{vest}^2 a_n^2 \Delta\right) \\
&= \sum_{n=0}^{N}\left(\mathrm{I}_{\delta x_{vis,n}}\left(sin(h)\right) + \mathrm{I}_{\delta x_{vest,n}}\left(sin(h)\right)\right).
\end{aligned}
\tag{19}
$$

In the above, the first line follows from re-expressing the posterior Eq. (11) in terms of $X_{comb}(T)$ (effectively turning it back into a likelihood) and computing its Fisher Information, the second line is based on substituting Eqs. (10) and (14) for k_{comb}^2 and $D(T)$, the third line utilizes the definitions of $A(T)$ and $V(T)$ in Eq. (13), and the last line uses the expression for Fisher Information of the momentary evidence, as discussed in the Section Momentary Evidence.

To relate this kind of optimality to that of standard diffusion models, let us consider how it compares to that of the Sequential Probability Ratio Test (SPRT, (Wald 1947; Wald and Wolfowitz 1948)), of which this diffusion model is a continuous-time implementation (Bogacz, Brown et al. 2006). The SPRT assumes that the likelihood function associated with either option (e.g. “left” and “right”) is known and time-invariant, and consists of accumulating the log-likelihood ratios of the momentary evidences to form a log-posterior. Once this log-posterior reaches a lower or upper time-invariant threshold (in units of log-odds, and thus belief/error rate), the more likely option is chosen. This procedure has been shown to be optimal in at least two senses (Wald and Wolfowitz 1948; Bogacz, Brown et al. 2006). First, if the assumptions of the SPRT are satisfied, it performs Bayes-optimal accumulation of evidence. Second, of all fixed or sequential sample tests that feature the same or lower error rate as the SPRT, the SPRT is the procedure that leads to the fastest decisions, on average.

Our decision procedure features the same optimality guarantees as the SPRT in the first sense that it is Bayes-optimal. However, it follows different underlying assumptions about the momentary evidence and how decisions are made, such that the second form of optimality relating to the speed of the decisions is not applicable. In particular, and in contrast to the SPRT, the likelihood function we utilize contains a nuisance parameter (the heading magnitude $|h|$) that we integrate out to find the belief

(e.g., in the simplest case Eq. (4)). A consequence is that the belief at decision time, and thus the error rate, is, unlike in the SPRT, not time-invariant (see Eq. (5)). As this error rate is a function of the decision time, it cannot be fixed, such that we cannot compare the decision speed of our procedure to that of others with the same error rate. Alternatively, we could have attempted to operate with the error rate averaged over decision times. However, given that there exists no analytical expression for the decision time distribution and that the problem cannot be addressed by the same means as the SPRT (using Wald's Martingale), this approach is unlikely to yield analytical statements. For a recent related attempt that uses numerical rather than analytical means, see (Drugowitsch, Moreno-Bote et al. 2012).

The model's psychometric function and discrimination threshold

To provide better insight into how the different model components contribute to its performance, we derive the model's psychometric function and discuss how it relates to the heading discrimination threshold. Considering for now the unimodal case with time-varying reliability, the psychometric function is formed by plotting how the fraction of choosing one of the two options changes as a function of heading, h (or, equivalently, its sine, $\sin(h)$). With respect to diffusion models, this fraction is the probability $p\left(X(T)=\theta \mid \sin(h)=H, T, X(T)=\pm\theta\right)$ for some heading H, and at some decision time T at which a boundary has been reached (i.e. $X(T)=\pm\theta$), that the particle has reached the upper boundary, $X(T)=\theta$.

We find this probability by first relating it to the model's posterior, conditional on the heading magnitude $|h|$. Specifically, due to the symmetric nature of our task (both responses are a-priori equally likely correct) and the symmetry of our model (inverting the sign of both evidence and responses leaves the behavior unchanged), it can be shown (see Eq. (17) in (Drugowitsch, Moreno-Bote et al. 2012)) that the model's choice probability equals its posterior belief, that is

$$p\left(X(T)=\theta \mid \sin(h)=H, T, X(T)=\pm\theta\right)=p\left(\sin(h)=H \mid X(T)=\theta, T, \sin(h)=\pm H\right). \quad (20)$$

In the above, the term on the right-hand side is the model's posterior that, given that the upper bound was reached at time T, the heading magnitude was $\sin(h)=H$ rather than $\sin(h)=-H$. This posterior is found by restricting Eq. (6) to these two cases, resulting in

$$p(\sin(h)=H \mid X(T)=\theta, T, \sin(h)=\pm H) \propto e^{Hk\theta-\frac{1}{2}H^2k^2B(T)},$$
$$p(\sin(h)=-H \mid X(T)=\theta, T, \sin(h)=\pm H) \propto e^{-Hk\theta-\frac{1}{2}H^2k^2B(T)}. \quad (21)$$

Using Eq. (20) and the fact that the probabilities in Eq. (21) sum to 1, we find after some cancellation of terms that the psychometric function is given by the logistic sigmoid,

$$p\left(X(T)=\theta \mid \sin(h)=H, X(T)=\pm\theta\right)=\frac{1}{1+e^{-2kH\theta}}. \quad (22)$$

Note that the above is increasing in k, H, and θ, as one would intuitively expect. Furthermore, it is independent of decision time T. Thus, as k and θ determine the slope of this function around $H=0$, the psychometric curve's steepness around this point grows with both the subject's sensitivity and the height of the bound. As we have shown the multimodal case to be reducible to a single diffusion model, it features the same psychometric function. A similar psychometric curve for diffusion models with constant reliability over time can be derived by the use of Wald's Martingale (see, for example, (Shadlen, Hanks et al. 2006) for a derivation). This derivation can be generalized to the time-variant reliability case, leading again to Eq. (22).

To relate the model's psychometric curve to the heading discrimination threshold, we assume that the latter is determined by fitting a cumulative Gaussian $\Phi\left(\frac{H}{\sigma}\right)$ with discrimination threshold σ to this psychometric function. Furthermore, we note that the logistic sigmoid $\left(1+\exp(-\beta H)\right)^{-1}$ and the above cumulative Gaussian are cumulative distribution functions of the zero-mean Logistic distribution $\text{Logistic}\left(0,\beta^{-1}\right)$ with scale β^{-1} and the zero-mean Gaussian $\text{N}\left(0,\sigma^2\right)$ with variance σ^2, respectively, evaluated at H. These two functions are closely matched by equating the variances of their underlying random variables, which results in $\sigma \approx \frac{\pi}{\sqrt{3}\beta}$. Thus, if we use $\beta=2k\theta$ from Eq. (22), we find that the discrimination threshold resulting from the extended diffusion model is approximately

$$\sigma \approx \frac{\pi}{\sqrt{12}k\theta}, \tag{23}$$

that is, it is inversely proportional to the sensitivity k and the bound height θ.

Model Parameterization and Fitting

In the previous sections we have described the Bayes-optimal decision model for both the combined and the unimodal conditions. Here, we show how we have fitted these models to behavioral data obtained from human subjects. We first describe the model parametrization and then describe how we have found the parameters, separately for each subject, that best explain the behavior. Finally, we describe a set of alternative, sub-optimal models that we have proposed in the main text as alternative hypotheses of how the observed behavior was generated.

Model Parameterization

The reliability of the visual cue was controlled by the percentage of dots that moved according to the current heading direction, from one video frame to the next, rather than being relocated randomly within the 3D volume. This percentage, called the motion coherence c, remained constant within a trial, but changed between trials, taking values $\{25\%, 37\%, 70\%\}$ ($c \in \{0.25, 0.37, 0.70\}$) ($\{0\%, 12\%, 25\%, 37\%, 51\%, 70\%\}$ for subjects B2, D2, F2). The subject's sensitivity to the momentary visual evidence depends on coherence, such that $k_{vis}(c)$ is a function of c. In the main text we lay out an argument, based on neurophysiological evidence, for how we believe coherence influences the sensitivity to the visual cue. Critically, this argument leads to the assumption that a change in c not only modifies the drift rate in the diffusion model, according to $k_{\sigma,vis}(c) \propto a_{vis} c^{\gamma_{vis}}$ (a_{vis} and γ_{vis} being model parameters), but also causes the diffusion variance to change according to $\sigma^2(c) \propto 1 + b_{vis} c^{\gamma_{vis}}$ (where b_{vis} is another model parameter). The decision bound $\theta_{\sigma,vis}$, on the other hand, cannot depend on coherence, as the latter is unknown to the subject. Specifying the visual-only conditions by drift rate, diffusion variance, and bound would lead to over-parameterization, as a model with diffusion variance different from unity generates the same behavior as a model that has

unit diffusion variance and drift rate and bounds adequately normalized (see above). We avoid over-parameterization by performing this normalization, resulting in

$$k_{vis}(c) = \frac{k_{\sigma,vis}(c)}{\sigma(c)} = \frac{a_{vis} c^{\gamma_{vis}}}{\sqrt{1 + b_{vis} c^{\gamma_{vis}}}}, \quad \theta_{vis}(c) = \frac{\theta_{\sigma,vis}}{\sigma(c)} = \frac{\theta_{\sigma,vis}}{\sqrt{1 + b_{vis} c^{\gamma_{vis}}}}. \tag{24}$$

This allows for an arbitrary proportionality constant in the relationship $\sigma^2(c) \propto 1 + b_{vis} c^{\gamma_{vis}}$, as this constant can be absorbed into a_{vis} and $\theta_{\sigma,vis}$. To summarize, we model behavior in the visual condition for any coherence by a unit variance diffusion model with sensitivity time-course given by $v(t)$, and parameterized by $\{a_{vis}, \gamma_{vis}, b_{vis}, \theta_{\sigma,vis}\}$.

In the vestibular condition, momentary evidence is not influenced by coherence, such that we can model behavior with a unit-variance diffusion model having sensitivity k_{vest}, time-course $a(t)$, and a diffusion model bound θ_{vest}. Thus, the model for the vestibular condition is parameterized by $\{k_{vest}, \theta_{vest}\}$.

In the combined condition, the coherence of the visual stimulus again influences the sensitivity to the momentary evidence. Given that visual and vestibular cues are combined optimally, the sensitivity to the evidence is completely determined by that to the two separate cues. In particular, we have $k_{comb}(c)^2 = k_{vis}(c)^2 + k_{vest}{}^2$ by Eq. (10) and its sensitivity time-course $d(t,c)$ given by Eq. (16) with k_{vis} replaced by $k_{vis}(c)$. As in the visual condition, we assume a constant bound $\theta_{\sigma,comb}$ and a variance that is linearly related to coherence taken to power γ_{comb}, such that the normalized bound becomes

$$\theta_{comb}(c) = \frac{\theta_{\sigma,comb}}{\sqrt{1 + b_{comb} c^{\gamma_{comb}}}}. \tag{25}$$

Thus, the model for the combined condition is characterized by a unit-variance diffusion model with sensitivity determined by the unimodal conditions, and a bound that is parameterized by $\{\theta_{\sigma,comb}, b_{comb}, \gamma_{comb}\}$.

We assume that reaction times featured by the subjects are composed of the decision time, as predicted by the diffusion model, and a non-decision time that captures the initial stimulus processing delay and the motor preparation time. We require this non-

decision time to be constant for all stimuli within each of the three stimulus modalities (vestibular, visual, combined), but we allow it to vary between modalities. Thus, the non-decision time is captured by the three parameters $\{t_{nd,vis}, t_{nd,vest}, t_{nd,comb}\}$. To account for random choices due to accidental button presses or lapses of attention, we introduce a lapse probability p_{lapse} with which the decision was performed randomly (with probability $1/2$ for each motion direction) rather than as predicted by the diffusion model. Additionally, we captured a potential bias in heading perception (i.e., horizontal shift of the psychometric function) by one additional bias parameter $\tilde{h}_{c,cond}$ for each combination of stimulus modality and coherence. Overall, given that the diffusion model predicts mean decision times represented by $t_{DM,corr}(h,c,cond,\varphi)$ and $t_{DM,incorr}(h,c,cond,\varphi)$ for correct and incorrect decisions, respectively, with model parameters φ, and given that the probability of choosing 'rightward' for each combination of heading direction h, visual motion coherence $c \in \{0.25, 0.37, 0.70\}$ ($c \in \{0, 0.12, 0.25, 0.37, 0.51, 0.70\}$ for subjects B2, D2, F2) and stimulus condition $cond \in \{vis, vest, comb\}$ is represented by $p_{DM,r}(h,c,cond)$, we assumed that the subject would feature mean reaction times and choice probabilities given by

$$
\begin{aligned}
t_{corr}(h,c,cond,\varphi) &= t_{DM,corr}\left(h+\tilde{h}_{c,cond}, c, cond, \varphi\right) + t_{nd,cond}, \\
t_{incorr}(h,c,cond,\varphi) &= t_{DM,incorr}\left(h+\tilde{h}_{c,cond}, c, cond, \varphi\right) + t_{nd,cond}, \\
p_r(h,c,cond,\varphi) &= (1-p_{lapse})\, p_{DM,r}\left(h+\tilde{h}_{c,cond}, c, cond, \varphi\right) + p_{lapse}\tfrac{1}{2}.
\end{aligned}
\tag{26}
$$

The diffusion model itself and the non-decision times were parameterized by 12 parameters $\{a_{vis}, \gamma_{vis}, b_{vis}, \theta_{\sigma,vis}, k_{vest}, \theta_{vest}, \gamma_{comb}, b_{comb}, \theta_{\sigma,comb}, t_{nd,vis}, t_{nd,vest}, t_{nd,comb}\}$, and an additional 8 parameters (14 parameters for subjects B2, D2, F2) captured the biases and lapse rates. With these parameters, we modeled reaction times (separately for correct and incorrect decisions) and proportions of rightward/leftward choices for 56 different combinations of heading direction h, coherence c, and stimulus condition $cond$. In total, 168 data points (312 data points for subjects B2, D2, F2) were fit with a model

containing 12 primary parameters (along with 8 or 14 additional parameters to account for biases and lapse rates).

Alternative Parameterization

To ensure that our particular choice of parameterization did not bias our results on optimal evidence accumulation, we performed the same analysis with two additional parametric forms for sensitivities and normalized bounds. As shown in Fig. 7-figure supplement 2b and 2c, neither form changed our conclusions. Furthermore, Bayesian model comparison indicated that these alternative forms introduce a larger number of parameters than justifiable by the improvement in goodness-of-fit (Fig 4a).

The first alternative parameterization questions the relation between diffusion variance and drift rate. For the visual condition in the optimal model we have assumed this variance to be proportional to $1+b_{vis}c^{\gamma_{vis}}$ and the drift to follow $a_{vis}c^{\gamma_{vis}}$. In both cases, coherence is take to the same power γ_{vis}. To test if a different power might explain the behavior better, we left the drift rate unchanged, but modified the variance to be proportional to $1+b_{vis}c^{\xi_{vis}}$, where ξ_{vis} is an additional parameter. Figure 3-figure supplement 2 reveals that this modification leads to slightly different fits (dashed lines), while not qualitatively changing the relation between a model assuming optimal evidence accumulation and variants that do not (Fig. 7-figure supplement 2b). However, Fig. 7-figure supplement 2a shows that introducing this additional parameter is not justified by the minor increase in goodness-of-fit.

A second alternative parameterization abolishes any functional relationship between drifts, bounds, and coherences, and instead fits these drifts and bounds for each coherence and modality separately. That is, for the visual condition, drifts and bounds are modeled by one separate $k_{vis}(c)$ and $\theta_{vis}(c)$ per coherence. The vestibular condition is modeled, as before, with two parameters, k_{vest} and θ_{vest}. In the combined condition, we assume optimal cue combination, such that $k_{comb}(c)=\sqrt{k_{vis}(c)^2+k_{vest}{}^2}$, but fit the bounds $\theta_{comb}(c)$ for each coherence separately. Thus, the model still assumes optimal accumulation of evidence across both time and cues, but makes no statement about how the tradeoff between speed and accuracy depend on the visual coherence. It replaces the 9

parameters (not counting the non-decision times) of the original model by 11 parameters (20 parameters for subjects B2, D2, F2) for drifts and bounds. Figure 3-figure supplement 1 shows the drifts and bounds for full model fits for each subject, and how they relate the other two parameterizations. As shown in Fig. 7-figure supplement 2c, abolishing the function form for these model variables does not qualitatively change the relation between optimal and suboptimal models. However, they do not explain the behavior better than a model with the original parameterization (Fig. 7-figure supplement 2a).

Model Fitting

We fit the model separately to the behavior of each subject by finding the model parameters φ (see previous section) that maximized their likelihood given the observed behavior. As in (Palmer, Huk et al. 2005), we assumed that the fraction of correct choices followed a binomial distribution, and that the reaction times of correct and incorrect choices were distributed according to a Gaussian centered on the empirical mean and spread according to the standard error. That is, for each combination of heading h, coherence c, and condition $cond$, we assumed the likelihood of φ to describe the choice fraction by

$$L_{r,h,c,cond}(\varphi) = \mathrm{Bin}\left(\hat{p}_r(h,c,cond)\, n_{h,c,cond} \mid n_{h,c,cond}, p_r(h,c,cond,\varphi)\right), \qquad (27)$$

which is a Binomial distribution over the observed number of rightwards choices, $\hat{p}_r(h,c,cond)\, n_{h,c,cond}$, given a total number of $n_{h,c,cond}$ trials and the model prediction $p_r(h,c,cond,\varphi)$. The likelihood terms describing the reaction times were given by the Gaussian

$$L_{corr,h,c,cond}(\varphi) = \mathrm{N}\left(\hat{t}_{corr}(h,c,cond) \mid t_{corr}(h,c,cond,\varphi), \frac{\mathrm{var}_{corr}(h,c,cond,\varphi)}{n_{corr,h,c,cond}}\right), \qquad (28)$$

for reaction times corresponding to correct choices, and an analogous term $L_{incorr,h,c,cond}(\varphi)$ for those corresponding to incorrect choices. In the above $\hat{t}_{corr}(h,c,cond)$ is the observed mean reaction time over the $n_{corr,h,c,cond}$ trials in which correct choices were made, $t_{corr}(h,c,cond,\varphi)$ is the mean reaction time predicted by the model, and

$\mathrm{var}_{corr}\left(h, c, cond, \varphi\right)$ is the variance of this prediction. Overall, the complete likelihood was given by

$$L(\varphi) \prod_{h,c,cond} L_{r,h,c,cond}\left(\varphi\right) L_{corr,h,c,cond}\left(\varphi\right) L_{incorr,h,c,cond}\left(\varphi\right). \tag{29}$$

Fitting the model consisted of finding the parameter vector φ for each subject that maximized this likelihood.

Model predictions were found by evaluating Eq. (26). For each combination of heading, coherence, and stimulus condition, we computed the diffusion model predictions by numerically evaluating the reaction time distributions for either choice in steps of 5ms, using a method described previously (Smith 2000). Based on these distributions, we computed the probability of a choosing 'rightward' and the mean and variance of the reaction times for either choice.

To find the maximum likelihood parameters, we acquired a three-step approach that avoided getting stuck in likelihood function plateaus or local maxima. First, we performed gradient ascent on the log-likelihood to find the initial (potentially local) maximum. We used the found parameter vector as initial sample for taking 44000 samples from the Bayesian parameter posterior by Markov Chain Monte Carlo methods, assuming a bounded uniform parameter prior. Last, we picked the highest-likelihood sample as a starting point for another gradient ascent step to find the posterior's mode. This mode was used as the maximum likelihood parameter vector. The resulting model parameters are shown for each subject in Fig. 3-figure supplement 2. All pseudo-gradient ascent maximizations were performed with the Optimization Toolbox of Matlab R2013a (Mathworks), using stringent stopping criteria ($\mathrm{TolFun} = \mathrm{TolX} = 10^{-20}$) to prevent premature convergence. For posterior sampling we utilized a custom Matlab implementation of slice sampling (Neal 2003). The parameter posterior variances reported in Fig. 3-figure supplement 2 were computed from the second half of all posterior samples of the Markov Chain.

The coefficient of determination that was used to describe the overall goodness-of-fit in Fig. 7 was computed as follows. The average coefficient of determination $R^2(\varphi) = \frac{1}{2}\left(R^2_{psych}(\varphi) + R^2_{chron}(\varphi)\right)$ is for each subject the average of $R^2_{psych}(\varphi)$ and

$R^2_{chron}(\varphi)$, that is, the adjusted coefficients of determination for the psychometric and the chronometric curves, respectively. $R^2_{psych}(\varphi)$ is computed from

$$\tilde{R}^2_{psych}(\varphi) = 1 - \frac{\sum_{h,c,cond} w_{h,c,cond}\left(\hat{p}_r(h,c,cond) - p_r(h,c,cond,\varphi)\right)^2}{\sum_{h,c,cond} w_{h,c,cond}\left(\hat{p}_r(h,c,cond) - \overline{p}_r\right)^2}, \quad (30)$$

by $R^2_{psych}(\varphi) = \tilde{R}^2_{psych}(\varphi) - \left(1 - \tilde{R}^2_{psych}(\varphi)\right)\frac{k}{N_s - k - 1}$, where $\hat{p}_r(h,c,cond)$ and $p_r(h,c,cond,\varphi)$ are the same terms as in the above likelihood, $\overline{p}_r$ is the mean probability of choosing right over all trials, $w_{h,c,cond}$ is the fraction of trials with heading h, coherence c and condition $cond$, k is the number of model parameters, and N_s is the number of trials performed by subject s. For the chronometric curve we consider reaction times for both correct and incorrect choices by computing $R^2_{chron}(\varphi) = \tilde{R}^2_{chron}(\varphi) - \left(1 - \tilde{R}^2_{chron}(\varphi)\right)\frac{k}{N_s - k - 1}$ from

$$\tilde{R}^2_{chron}(\varphi) = 1 - \frac{\sum_{h,c,cond}\begin{pmatrix} w_{corr,h,c,cond}\left(\hat{t}_{corr}(h,c,cond) - t_{corr}(h,c,cond,\varphi)\right)^2 + \\ w_{incorr,h,c,cond}\left(\hat{t}_{incorr}(h,c,cond) - t_{incorr}(h,c,cond,\varphi)\right)^2 \end{pmatrix}}{\sum_{h,c,cond}\begin{pmatrix} w_{corr,h,c,cond}\left(\hat{t}_{corr}(h,c,cond) - \overline{t}\right)^2 + \\ w_{incorr,h,c,cond}\left(\hat{t}_{incorr}(h,c,cond) - \overline{t}\right)^2 \end{pmatrix}}, \quad (31)$$

where $\hat{t}_{corr}(h,c,cond,\varphi)$ and $\hat{t}_{incorr}(h,c,cond,\varphi)$ are again the same as in the likelihood, $\overline{t}$ is the mean reaction time over all trials, and $w_{corr,h,c,cond}$ and $w_{incorr,h,c,cond}$ are the fractions of correct and incorrect trials (out of all trials, such that $w_{h,c,cond} = w_{corr,h,c,cond} + w_{incorr,h,c,cond}$), respectively, that match $h,c,cond$.

Alternative, Sub-Optimal Models

We compared the fit quality of the optimal model to that of various, mostly sub-optimal models. These models are described below.

Free cue combination weights. In the optimal model, the sensitivity to the momentary evidence for the combined condition is determined by the sensitivities to the two separate

cues. In particular, $k_{comb}(c)^2 = k_{vis}(c)^2 + k_{vest}{}^2$ is assumed to hold. We introduced an alternative model in which $k_{comb}(c)$ is a free parameter for each coherence that is fitted independently of $k_{vis}(c)$ and k_{vest} to test two things: first, we were interested in comparing if the independently fit $k_{comb}(c)$ match those predicted from fits to the unimodal conditions. As discussed in the main text, this turns out to be the case (Fig. 6). Second, we wanted to know if loosening the optimality constraint explains the subjects' behavior better. For a fair comparison, we observe that this modification introduces one additional parameter per coherence when compared to the optimal model. Since the modified model is strictly more general than the optimal model, it is expected to fit the behavior at least as well or better than the optimal model. However, as described in the main text, Bayesian model comparison that takes into account the additional number of parameters revealed that the increased goodness-of-fit does not justify the additional degrees of freedom (Fig. 7).

Fixed cue combination weights. When performing optimal cue combination, the different cues ought to be weighted according to their respective sensitivities, as described by Eq. (17). We tested whether this was indeed the case by introducing a model variant that weights the momentary evidence of both cues equally. The evidence provided by each cue was still accumulated optimally over time according to Eq. (12), such that the momentary evidences were given by $\dot{X}_{vis}(t) = v(t)\dot{x}_{vis}(t)$ and $\dot{X}_{vest}(t) = a(t)\dot{x}_{vest}(t)$. However, rather than combining across modalities as given by Eq. (17), we performed a simple average: $\dot{X}_{comb}(t) = \frac{1}{2}\left(\dot{X}_{vis}(t) + \dot{X}_{vest}(t)\right)$. In the combined condition, this resulted in a diffusion model with drift rate given by $\frac{1}{2}\left(v(t)^2 k_{vis}(c) + a(t)^2 k_{vest}\right) sin(h)$ and diffusion variance given by $\frac{1}{4}\left(v(t)^2 + a(t)^2\right)$. The bound was left unchanged, as given by Eq. (25). The number of parameters for this model variant is the same as for the optimal model.

Weighting both cues by either acceleration or velocity. When designing the model we have assumed that the sensitivity time-course of the visual and vestibular modality is

determined by the motion velocity and acceleration, respectively. To test this choice we introduced a model variant that weights both modalities by either acceleration or velocity. For the first variant we replaced $v(t)$ in Eq. (12) and all equations that follow by $a(t)$. For the second variant we replaced $a(t)$ by $v(t)$ in all relevant equations.

No temporal weighting of momentary evidence. Our theory predicts that optimal accumulation of evidence over time requires this evidence to be weighted according to its associated momentary sensitivity, as given by Eq. (7). To test this, we introduced an additional model that did not perform this temporal weighting. Instead, we assumed the diffusion models for the unimodal conditions to feature a unit diffusion variance and un-weighted drift rates, $v(t)k_{vis}(c)sin(h)$ and $a(t)k_{vest}(c)sin(h)$, for the visual and vestibular conditions, respectively. The cues were still combined according to the optimal combination rule, Eq. (10), resulting in a diffusion model for the combined condition with unit variance and drift rate given by $\frac{k_{vis}{}^2 v(t)+k_{vest}{}^2 a(t)}{k_{comb}} sin(h)$. The bound was left unchanged, resulting in the number of parameters to be the same as for the optimal model.

No temporal weighting and fixed cue combination weights. The last model variant discards both the assumption of temporal weighting of evidence and the assumption of sensitivity-based weighting when combining the cues across modalities. Thus, the diffusion models describing the unimodal conditions featured, as before, a unit diffusion variance and drift rates, $v(t)k_{vis}(c)sin(h)$ and $a(t)k_{vest}(c)sin(h)$, for the visual and vestibular conditions, respectively. In the combined condition, momentary evidence was summed according to $\dot{x}_{comb}(t) = \frac{1}{\sqrt{2}}\left(\dot{x}_{vis}(t) + \dot{x}_{vest}(t)\right)$, resulting in a diffusion model with unit variance and drift rate $\frac{1}{\sqrt{2}}\left(v(t)k_{vis}(c) + a(t)k_{vest}\right)sin(h)$. The $\frac{1}{\sqrt{2}}$ weighting was chosen to ensure unit variance, but any other weighting would have resulted in the same fits. The bounds were parameterized as in the optimal model, such that the number of parameters was the same as in the original model.

References

Bogacz, R., E. Brown, et al. (2006). "The physics of optimal decision making: a formal analysis of models of performance in two-alternative forced-choice tasks." Psychological Review **113**(4): 700-765.

Clark, J. J. and A. L. Yuille (1990). Data fusion for sensory information processing systems. Boston, Kluwer Academic Publishers.

Drugowitsch, J., R. Moreno-Bote, et al. (2012). "The Cost of Accumulating Evidence in Perceptual Decision Making." J Neurosci **32**(11): 3612-3628.

Gold, J. I. and M. N. Shadlen (2002). "Banburismus and the brain: decoding the relationship between sensory stimuli, decisions, and reward." Neuron **36**(2): 299-308.

Kiani, R. and M. N. Shadlen (2009). "Representation of confidence associated with a decision by neurons in the parietal cortex." Science **324**(5928): 759-764.

Laming, D. R. J. (1968). Information theory of choice-reaction times. London,, Academic P.

Lisberger, S. G. and J. A. Movshon (1999). "Visual motion analysis for pursuit eye movements in area MT of macaque monkeys." J Neurosci **19**(6): 2224-2246.

Neal, R. M. (2003). "Slice sampling." Annals of Statistics **31**(3): 705-767.

Palmer, J., A. C. Huk, et al. (2005). "The effect of stimulus strength on the speed and accuracy of a perceptual decision." J Vis **5**(5): 376-404.

Price, N. S., S. Ono, et al. (2005). "Comparing acceleration and speed tuning in macaque MT: physiology and modeling." J Neurophysiol **94**(5): 3451-3464.

Ratcliff, R. (1978). "Theory of Memory Retrieval." Psychological Review **85**(2): 59-108.

Schlack, A., B. Krekelberg, et al. (2007). "Recent history of stimulus speeds affects the speed tuning of neurons in area MT." J Neurosci **27**(41): 11009-11018.

Shadlen, M. N., T. D. Hanks, et al. (2006). The Speed and Accuracy of a Simple Perceptual Decision: A Mathematical Primer. Bayesian Brain: Probabilistic Approaches to Neural Coding. K. Doya, S. Ishii, A. Pouget and R. P. N. Rao, MIT Press**:** 209-238.

Smith, P. L. (2000). "Stochastic Dynamic Models of Response Time and Accuracy: A Foundational Primer." J Math Psychol **44**(3): 408-463.

Wald, A. (1947). Sequential analysis. New York, London, J. Wiley & sons, Chapman & Hall.

Wald, A. and J. Wolfowitz (1948). "Optimum Character of the Sequential Probability Ratio Test." The Annals of Mathematical Statistics **19**(3): 326-339.
