# Supplementary material for: Optimal multisensory decision-making in a reaction-time task
Source: eLife. 2014 Jun 14;3:e03005. doi: 10.7554/eLife.03005 (PMC4102720; doi:10.7554/eLife.03005)
Supplement: Supplementary file 2. — Outcome of additional statistical hypothesis tests. DOI: http://dx.doi.org/10.7554/eLife.03005.016 [file elife03005s002.pdf]

Optimal multisensory decision-making in a reaction-time task

Outcome of additional statistical hypothesis tests

Jan Drugowitsch, Gregory C. DeAngelis, Eliana M. Klier,
Dora E. Angelaki, Alexandre Pouget

Subject	$\sigma_{comb}(c) < \min\{\sigma_{vis}(c), \sigma_{vest}\}$						$\sigma_{comb}(c) > \sigma_{vis}(c)$						$\sigma_{comb}(c) > \sigma_{pred}(c)$						$\sigma_{comb}(c) < \sigma_{vest}$
	0%	12%	25%	37%	51%	70%	0%	12%	25%	37%	51%	70%	0%	12%	25%	37%	51%	70%	70%
A			1	1		1			1	0.002		0			1	0		0	0
B			1	1		1			1	0		0			0.287	0		0	0
C			1	1		1			1	**1**		**1**			1	**1**		**0.168**	**0.210**
D			0.57	1		1			1	0		0			1	0		0	0
E			1	1		1			1	0.027		0			1	0		0	0.007
F			1	1		1			1	**0.662**		0.006			1	0		0	0
G			1	1		1			**0.005**	0		0			0	0		0	0
B2	1	1	1	1	1	1	1	1	1	**0.696**	0	0	1	1	0.072	0	0	0	0.039
D2	1	1	1	1	1	1	1	1	1	**1**	0	0	1	0.377	0.813	0.007	0	0	0
F2	1	1	1	1	1	1	1	1	1	0	0	0.042	0.319	0.325	0	0	0	0	0

Table A. p-values for comparisons of decision thresholds, for different coherence values of the visual stimulus. The performed tests are indicated in the first row (vis = visual-only condition, $vest$ = vest-only condition, $comb$ = combined condition, $pred$ = predicted threshold, c = coherence). From left to right, the four tests conducted are: i) whether the combined threshold is significantly less than the threshold of the more sensitive modality, ii) whether the combined threshold is significantly greater than the visual threshold, iii) whether the combined threshold is significantly greater than the predicted threshold, and iv) whether the combined threshold is significantly less than the vestibular threshold. The p-values in bold indicate deviations from the general effects reported in the main text. The p-values are computed from 5000 bootstrapped samples for each threshold, and are Bonferroni-corrected for multiple one-tailed comparisons. $p=0$ represents $p<0.0001$

Subject	25% coherence				37% coherence				70% coherence			
	0.69°	1.96°	5.6°	16°	0.69°	1.96°	5.6°	16°	0.69°	1.96°	5.6°	16°
Miller’s bound violations (bold = p<0.05)												
A	1	0.9473	0.9867	1	1	1	0.9996	0.9317	1	1	1	0.9978
B	1	1	1	1	1	1	1	1	1	1	1	1
C	0.8943	1	0.9537	0.7310	0.9681	0.8782	1	1	0.9250	1	0.9749	0.9973
D	0.5775	0.9606	0.9553	0.9958	1	1	0.9933	0.9919	1	0.9952	1	0.2127
E	1	1	0.6683	0.9159	0.9928	0.9839	0.9840	0.9840	1	1	1	1
F	0.8853	0.7483	1	0.8086	0.9655	0.9910	1	0.9674	0.9896	0.6454	0.9986	0.0890
G	0.9335	0.9777	0.6540	0.4549	0.7447	0.9394	0.8502	0.4544	0.9927	0.9182	0.9344	0.0358
Grice’s bound violations (bold = p<0.05)												
A	**0.0001**	**0.0346**	**0**	0.0701	**0**	**0.0025**	**0.0361**	**0.0373**	**0.0102**	**0.0189**	**0.0005**	0.1195
B	**0**	**0**	**0**	**0**	**0**	**0**	**0**	**0**	**0**	**0**	**0.0007**	**0**
C	**0.0070**	**0.0119**	**0.0398**	**0.0016**	0.8851	**0.0267**	0.2593	**0**	**0**	**0**	**0**	**0**
D	**0.0002**	**0**	**0.0138**	**0.0050**	**0**	**0**	**0.0171**	**0.0158**	**0**	**0**	0.1867	0.9706
E	**0.0094**	**0.0394**	**0.0039**	0.0750	**0.0002**	**0.0080**	**0.0377**	**0.0078**	**0**	**0**	**0**	**0.0016**
F	0.5889	0.4669	0.2710	**0.0090**	0.1625	0.3408	0.0671	0.0878	0.6905	0.7897	0.8045	1
G	**0.0002**	0.0823	0.1451	0.1495	**0.0199**	**0.0116**	**0.0053**	**0.0378**	**0**	**0**	**0**	0.9364

Table B. Comparing observed reaction time distributions to those predicted by a parallel race model, subjects A-G. Parallel race models predict the reaction time distribution in the combined condition, based on those observed in the two unimodal conditions. We tested for all heading/coherence combinations if these predictions matched the observed behavior. A violation of Miller’s bound (Miller 1982) or Grice’s bound (Grice, Canham et al. 1984) implies that the subject reacted significantly faster or slower, respectively, than predicted by a parallel race model. The table shows the uncorrected p-values resulting from a one-sided two-sample Kolmogorov-Smirnov test between the observed distributions and those corresponding to either of the two bounds. Values in boldface are significant bound violations, based on a Bonferroni-corrected threshold at 0.05.

Subject	0.69°	1.96°	5.6°	16°	0.69°	1.96°	5.6°	16°	0.69°	1.96°	5.6°	16°
Miller's bound violations (bold = p<0.05)												
	0% coherence				12% coherence				25% coherence			
B2	0.1007	0.3100	0.2449	0.2197	0.7388	0.7375	0.5766	0.4710	0.5262	0.7974	0.8567	0.4561
D2	0.9966	0.9701	0.9573	0.9213	0.9968	0.9892	0.9908	0.9964	0.9302	0.9888	0.9933	0.8156
F2	1	1	1	1	0.9969	0.9974	0.9978	0.9981	1	1	1	1
	37% coherence				51% coherence				70% coherence			
B2	0.7311	0.9129	0.9245	0.8675	0.5534	0.7226	0.7937	0.4994	0.1135	0.4465	0.3904	0.1012
D2	1	1	1	0.9890	1	1	0.9977	0.9963	1	1	1	1
F2	1	1	1	1	0.9515	0.9592	0.9652	0.7781	0.1522	0.2088	0.2094	0.0043
Grice's bound violations (bold = p<0.05)												
	0% coherence				12% coherence				25% coherence			
B2	0.5949	0.6636	0.6041	0.5912	0.5061	0.2075	0.0961	0.1455	0.1108	0.0781	0.0162	0.0408
D2	**0**	**0**	**0**	**0**	0.0012	**0**	**0**	**0**	**0**	**0**	**0**	**0**
F2	**0.0030**	**0.0006**	**0**	**0**	**0.0004**	**0**	**0**	**0**	**0.0033**	**0.0020**	**0.0001**	**0.0003**
	37% coherence				51% coherence				70% coherence			
B2	0.0619	0.0201	0.0106	0.0093	0.7827	0.4873	0.1659	0.3565	0.0697	**0.0012**	**0.0008**	0.0044
D2	**0**	**0**	**0**	**0**	**0**	**0**	**0**	**0**	**0**	**0**	**0**	**0**
F2	0.0153	0.0106	**0.0004**	**0.0002**	0.0083	0.0061	**0.0028**	0.0179	0.9530	0.9137	0.9257	0.9347

Table B (continued). Comparing observed reaction time distributions to those predicted by a parallel race model, subjects B2, D2, F2. See previous page for details.

Subject	2-way ANOVA					JB	Friedman test		
	factor	df	df(err)	F	p		df	χ^2	p
A	mod	1	1636	1167.45	0	7	1	467.07	0
	head	3		47.00	0				
	int	3		26.26	0				
B	mod	1	2867	1847.41	0	5	1	706.80	0
	head	3		204.96	0				
	int	3		47.61	0				
C	mod	1	1805	450.91	0	7	1	347.14	0
	head	3		23.34	0				
	int	3		3.71	0.011				
D	mod	1	4320	1720.95	0	8	1	935.21	0
	head	3		316.96	0				
	int	3		107.02	0				
E	mod	1	3254	2536.39	0	5	1	872.80	0
	head	3		126.10	0				
	int	3		62.97	0				
F	mod	1	2691	41.64	0		1	35.77	0
	head	3		167.17	0				
	int	3		8.63	0				
G	mod	1	3074	1459.71	0	7	1	565.61	0
	head	3		388.60	0				
	int	3		115.67	0				
B2	mod	1	1419	1428.33	0	2	1	604.62	0
	head	3		211.32	0				
	int	3		113.57	0				
D2	mod	1	1786	2214.62	0	5	1	792.66	0
	head	3		308.53	0				
	int	3		127.79	0				
F2	mod	1	1428	67.64	0	8	1	87.26	0
	head	3		171.53	0				
	int	3		11.39	0				

Table C. Statistical tests applied to comparing reaction times between 70%-coherence visual-only condition and vestibular-only condition. 2-way ANOVA: factors are modality (mod; 70% visual vs. vestibular), heading direction (head), and interaction (int); df(err) = df of error term; F = F statistics; p = p-value (p=0 represents $p<0.0001$). The JB column reports the number of violations (out of 8; 4 x visual, 4 x vestibular) of the normality assumption (Jarque-Bera, $p<0.05$). All tests are performed on the natural logarithm of the reaction time. These tests confirm that, for all subjects, the reaction times differ significantly between the visual and vestibular conditions. Except for subject C, who responded significantly slower, all subjects responded faster in the vestibular condition than in the 70% coherence visual condition (see Figure 3-figure supplement 1)

References

Grice, G. R., L. Canham, et al. (1984). "Combination rule for redundant information in reaction time tasks with divided attention." Percept Psychophys **35**(5): 451-463.

Miller, J. (1982). "Divided attention: evidence for coactivation with redundant signals." Cogn Psychol **14**(2): 247-279.
